# Field strength dependence, physiologic correlates, and prognostic significance of ventricular blood pool T2 mapping on cardiovascular magnetic resonance imaging^☆^

**DOI:** 10.1016/j.jocmr.2026.102760

**Published:** 2026-06-06

**Authors:** Moran Drucker Iarovich, Fadi Ibrahim, Joao Francisco Matos, Nilushi de Silva, William Holden Lowes, Yasbanoo Moayedi, Paaladinesh Thavendiranathan, Tilman Emrich, Akos Varga-Szemes, Kate Hanneman

**Affiliations:** a Joint Department of Medical Imaging, Toronto General Hospital, University Health Network (UHN), University of Toronto, Toronto, Canada; b Department of Medical Imaging, Temerty Faculty of Medicine, University of Toronto, Toronto, Canada; c Division of Cardiology, Peter Munk Cardiac Centre, Toronto General Hospital, University Health Network (UHN), University of Toronto, Toronto, Canada; d Department of Diagnostic and Interventional Radiology, University Medical Center of the Johannes Gutenberg-University Mainz, Langenbeckst, Mainz, Germany; e German Center for Cardiovascular Research (DZHK), Partner Site Rhine-Main, Mainz, Germany; f Department of Radiology and Radiological Science, Medical University of South Carolina, Charleston, South Carolina, USA

**Keywords:** Cardiovascular magnetic resonance, magnetic resonance imaging (MRI), Cardiomyopathy, Blood oxygenation, T2 mapping, parametric mapping

## Abstract

**Background and Purpose:**

Cardiovascular magnetic resonance (CMR) T2 mapping is sensitive to blood oxygenation. The purpose was to evaluate determinants of the right-to-left ventricular (RV/LV) blood pool (BP) T2 ratio, determine field strength-specific cut-points for prediction of major adverse cardiac events (MACE), and assess physiologic correlates.

**Methods:**

Adults undergoing CMR (at 1.5T or 3T) for evaluation of cardiomyopathy between 2018 and 2020 were retrospectively evaluated along with healthy controls. RV and LV BP T2 values were measured on a mid-ventricular short-axis T2 map with calculation of the RV/LV BP T2 ratio. Reproducibility was assessed in a random subset of 100 patients. Associations with cardiopulmonary exercise testing (CPET) parameters and major adverse cardiac events (MACE) were evaluated using Spearman correlation, Cox regression, and receiver operating characteristic analysis.

**Results:**

A total of 718 subjects were included: 678 with cardiomyopathy (mean age 50±16 years; 58% men) and 40 controls (mean age 45±15 years; 55% men). Over a median follow-up of 3.2 years (IQR 2.3–3.8), 53 patients experienced MACE. RV/LV BP T2 ratios were 18%–31% higher at 1.5T versus 3T (cardiomyopathy: 0.77±0.14 vs 0.59±0.13, p<0.001; controls: 0.79±0.11 vs 0.67±0.09, p<0.001) with excellent reproducibility (intra-observer ICC 0.96 and inter-observer ICC 0.95). Each 0.1-unit decrease in RV/LV BP T2 ratio was associated with 41%–42% increased hazard of MACE in multivariable analysis (HR 0.59, 95%CI 0.45, 0.78, p<0.001 at 1.5T and HR 0.58, 95%CI 0.40, 0.85, p=0.005 at 3T). Optimal cut-points for prediction of MACE were <0.70 at 1.5T (AUC 0.727) and <0.55 at 3T (AUC 0.689). Absolute RV BP T2 demonstrated similar prognostic performance. Among patients with CPET (n=150), lower RV/LV BP T2 ratios correlated significantly with reduced percent-predicted oxygen uptake (VO_2_), impaired heart rate recovery, and worse ventilatory efficiency (VE/VCO₂) at anaerobic threshold.

**Conclusions:**

The RV/LV BP T2 ratio reflects clinically meaningful cardiopulmonary physiology and independently predicts MACE, but is strongly influenced by MRI field strength, challenging assumptions of inherent field-independence and necessitating field strength-specific thresholds. Absolute RV BP T2 demonstrated comparable prognostic performance and may serve as a practical complementary metric. These findings establish RV BP T2 metrics as promising biomarkers for improved risk stratification in clinical CMR.

## Graphical abstract

**Figure:**
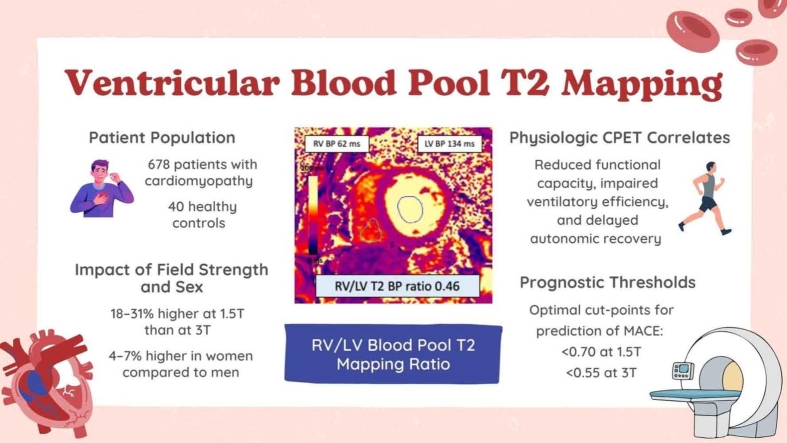

## 1 Background

Cardiovascular magnetic resonance (CMR) T2 mapping enables quantitative assessment of myocardial T2 relaxation times and is used to detect myocardial edema and inflammation [1], [2], [3]. CMR T2 values also correlate positively with blood oxygenation levels [4], [5]. The presence of deoxygenated hemoglobin shortens T2, a phenomenon known as the blood oxygenation level-dependent (BOLD) effect. This property has been extensively leveraged in functional brain MRI to assess regional neuronal activity through changes in blood flow and oxygenation via neurovascular coupling [6]. This same principle can be applied to the intracardiac blood pool (BP) [7].

Right ventricular (RV) BP T2 values are lower than left ventricular (LV) BP T2 values due to lower oxygenation of venous blood compared with systemic arterial blood [8]. RV blood pool T2 is further reduced in conditions associated with systemic desaturation, such as pulmonary hypertension and heart failure, and increased in the setting of left-to-right shunts, including atrial and ventricular septal defects [8], [9], [10].

Absolute T2 values are influenced by multiple technical factors, including magnetic field strength, scanner type, and sequence [11]. As a result, direct T2-based quantification of blood oxygenation is challenging. Proposed approaches to address this variability include scanner-specific calibration and multiparametric acquisitions that require time-consuming modeling and post-processing [5], [12], [13].

To mitigate some of this variability, the ratio of RV to LV BP T2 (RV/LV BP T2) has been proposed as a non-invasive marker of intracardiac oxygenation gradients, with the assumption that normalization to LV values may reduce technical variability and improve comparability across platforms [8], [10], [14]. Prior studies have demonstrated associations between lower RV/LV BP T2 ratios and adverse cardiac outcomes, as well as correlations with functional capacity in patients with heart failure [10], [15], [16]. However, the extent to which this ratio is robust to key technical factors, particularly MRI field strength, has not been systematically evaluated. Additionally, data on sex-based differences, optimal prognostic cut-points, and associations with physiologic measures, such as cardiopulmonary exercise testing (CPET), remain limited. CPET assesses respiratory and cardiac responses to exercise, informing prognosis and the need for cardiac interventions in patients with heart failure [17].

The purpose of this study was to evaluate the impact of field strength and patient sex on RV/LV BP T2 ratio, determine field strength-specific cut-points for predicting major adverse cardiac events (MACE), and assess physiologic CPET correlates of this metric in patients undergoing CMR for evaluation of cardiomyopathy.

## 2 Methods

### 2.1 Study design and cohort

The institutional research ethics committee approved this single-center retrospective cohort study, and the requirement for written informed consent was waived. Consecutive adult patients (≥18 years of age) who had undergone clinically indicated cardiac MRI for evaluation of ischemic and non-ischemic cardiomyopathy at a large tertiary referral hospital network between January 2018 and February 2020 were included. A cohort of healthy controls was also included with no known cardiovascular disease, no cardiovascular risk factors, and normal CMR. Exclusion criteria were the inability to analyze LV or RV blood pool on CMR T2 maps, lack of clinical follow-up, and presence of congenital heart disease, including shunt physiology (ruled out based on prior echocardiography). A separate cohort of 15 health controls who underwent T2 mapping with both steady-state free precession (SSFP) and Fast Low Angle Shot (FLASH) readouts at 3T was included to compare T2 mapping pulse sequences.

### 2.2 CMR technique

CMR studies for both patients and controls were performed using 1.5 or 3 Tesla scanners (Magnetom AVANTOfit/SKYRAfit; Siemens Healthineers, Erlangen, Germany) using commercially available cardiac surface coils. Field strength was determined by scanner availability and scheduling workflow, not by patient characteristics. The protocol included long-axis and a stack of short-axis balanced cine steady-state free precession (SSFP) slices acquired with complete ventricular coverage (slice thickness 8 mm and 2 mm inter-slice gap). Mid-ventricular short-axis T1 and T2 mapping slices were acquired using a modified Look-Locker inversion recovery technique for native T1 mapping (5(3)3 inversion grouping and a matching T2 map using a T2-prep technique with read-out varying with external field-strength (balanced steady-state free precession [SSFP] at 1.5T and Fast Low Angle Shot [FLASH] at 3T) (Supplemental Table S1) [18], [19]. After application of inline motion correction algorithms, pixel-based T1 and T2 maps were automatically generated on the scanner. Long- and short-axis late gadolinium enhanced (LGE) images acquired using a 2D phase sensitive inversion recovery technique starting 12 min after administration of intravenous contrast (0.15 mmol/kg body weight of gadobutrol, Bayer Healthcare, Leverkusen, Germany).

### 2.3 CMR analysis

CMR studies were analyzed independently by an experienced fellowship-trained observer (4 years CMR experience), blinded to all clinical information using commercially available tools (Circle CVI42; Circle Cardiovascular Imaging, Calgary, Alberta, Canada). Left ventricular volumes, function, and mass were measured using automated contour detection with manual correction if required, as per established standards [20]. The presence of LGE was evaluated visually. RV and LV blood pool T2 values were assessed using a region of interest on a single mid-ventricular short-axis slice with a minimum area of 1.5 cm^2^, avoiding all trabeculations, papillary muscles, and inflow artifact. RV/LV BP T2 ratio was calculated as the ratio of absolute RV to LV BP T2 values. Myocardial T1 and T2 values were assessed at the interventricular septum, avoiding the right ventricular insertion points and blood pool [2], [21]. To evaluate intra- and inter-observer variability of blood pool T2 values, a random subset of 100 patients was re-evaluated by the same observer after a minimum one-month interval and by a second fellowship-trained observer (with 2 years CMR experience), blinded to all identifying data and the results of the initial assessment.

### 2.4 Clinical data

Clinical data on patient demographics, cardiac risk factors, clinical outcomes, and hematocrit (within 1 week of CMR) were extracted from the electronic patient record. The primary endpoint of MACE was defined as a composite of cardiac death, resuscitated sudden cardiac death (SCD), appropriate implanted cardioverter defibrillator (ICD) shock, or hospital admission for decompensated heart failure. Cardiac death was defined as death resulting from acute myocardial infarction, sudden cardiac death, death due to heart failure, death due to stroke, and death due to other cardiovascular causes as the primary cause of death [22]. Resuscitated sudden cardiac death was defined as sudden unexpected arrest of presumed cardiac origin within one hour of the onset of symptoms that was successfully resuscitated. Appropriate ICD shock was defined as ventricular tachyarrhythmia leading to appropriate ICD discharge. Heart failure hospitalization was defined as an unscheduled hospital admission for a primary diagnosis of heart failure exceeding 24 h, requiring treatment specifically directed at heart failure. Patients without events were censored at the time of their last clinical follow-up. Only new adverse cardiac events from the time of CMR were considered.

### 2.5 Cardiopulmonary exercise testing

Clinically indicated CPET was performed in a subset of patients within one year of CMR and evaluated according to standard guidelines [23]. Standardized CPET was conducted by either cycle ergometer using a ramp protocol of 10 W per minute or by treadmill following the Bruce or Modified Bruce protocol. Once coupled to the calibrated metabolic cart (Lode Corival, Lode B.V., Groningen, the Netherlands), breath-by-breath metabolic data along with continuous 12-lead electrocardiogram (ECG) heart rate, serial systolic blood pressure, and diastolic blood pressure were recorded at rest, unloaded exercise, exercise, and recovery. Unloaded exercise consisted of 2-min warm-up period without resistance to eliminate the possibility of any premature hyperventilation that may be confounding the exercise gas exchange values and to estimate internal work [24]. During testing, patients were encouraged to exercise to their maximum, symptom-limited capacity, ideally for a test duration of 8–12 min [25]. All cardiorespiratory data were collected using MGC Diagnostics Ultima Series system (Minnesota, USA). Patients who achieved a respiratory exchange ratio (RER) > 1.1 were considered to have given a maximal volitional effort [24]. Breath-by-breath analysis was used to assess various maximal and submaximal CPET parameters. Normative data is based on the Wasserman-Hansen Formula [25]. Rating of perceived exertion (RPE) was recorded based on the Borg 6–20 scale. Key cardiopulmonary exercise test parameters are summarized in Supplemental Table S2.

### 2.6 Statistical analysis

Statistical analysis was performed using a commercially available software package, STATA v14.1 (StataCorp, College Station, Texas). A two-tailed p-value of <0.05 was considered statistically significant. Continuous variables were presented using mean and standard deviation (SD) or median and interquartile range (IQR). Categorical variables were described using numbers and percentages. All continuous data were tested for normal distribution using the Shapiro-Wilk test. Categorical variables were compared using Fisher's exact test. Continuous variables were compared using independent samples t-test or Wilcoxon rank sum test. Paired t-test was used to compare T2 mapping values between FLASH and SSFP-based T2 sequences in the group of controls who had undergone T2 mapping with both techniques. Spearman correlation was used to evaluate associations between continuous variables. Time-to-event survival analysis using Cox proportional hazard models was used to examine associations between ventricular BP T2 metrics and MACE. Multivariable models were adjusted for patient age, sex, LGE, and cardiomyopathy etiology. Fully adjusted models were also adjusted for hematocrit. The proportional-hazards assumption was evaluated using Schoenfeld residuals. The likelihood ratio test was used to compare nested models to assess incremental prognostic value. Kaplan-Meier survival curves were used to visualize cumulative event-free survival, and the log-rank test was used to compare the cumulative incidence of MACE. Optimal RV/LV BP T2 ratio cut points were established using receiver operating characteristic (ROC) curve analysis to maximize sensitivity and specificity, as determined by the Youden index. Intra- and inter-observer agreement were assessed using individual intra-class correlation coefficients (ICCs) with two-way random-effects models.

## 3 Results

### 3.1 Patient characteristics

**Fig. 1:**
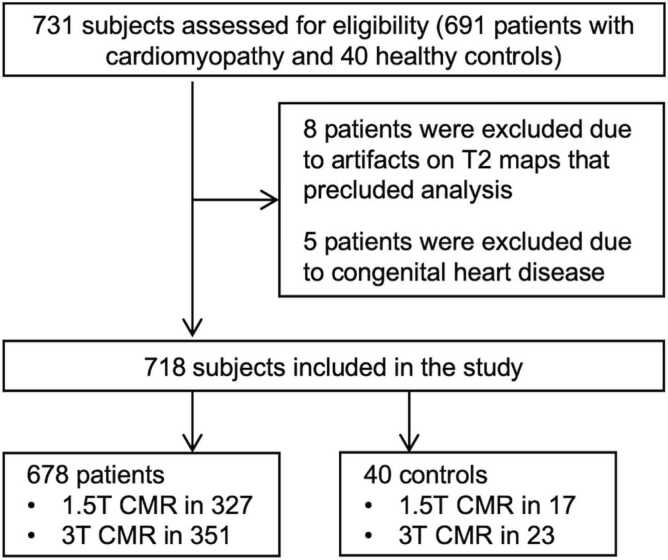
Subject flow diagram. *CMR* cardiovascular magnetic resonance

A total of 731 patients were evaluated for eligibility, 8 were excluded due to artifacts on T2 maps that precluded analysis and 5 were excluded due to congenital heart disease. The final study population included 718 subjects, 678 patients with cardiomyopathy (mean age 50±16 years; 58% men) and 40 healthy controls (mean age 45±15 years; 55% men), Table 1. Contemporary hematocrit was available in 504 patients (70%). Overall, 344 CMR studies were performed at 1.5T (48% overall; 327 patients and 17 controls) and 374 were performed at 3T (52% overall; 351 patients and 23 controls) (Fig. 1). Compared with controls, patients with cardiomyopathy had lower left ventricular ejection fraction (LVEF) (53±10% vs 58±3%, p=0.002) and right ventricular ejection fraction (RVEF) (51±10% vs 56±7%, p=0.05) and higher indexed LV mass (65 ± 23 g/m^2^ vs 53 ± 13 g/m^2^, p=0.003), Table 2.

**Table 1:** Patient demographics

	**All Patients** **(n=718)**	**Cardiomyopathy Patients** **(n=678)**	**Controls** **(n=40)**	**p-value**
Patient age (y)	50±16	50±16	45±15	0.06
Male, n (%)	413 (58%)	391 (58%)	22 (55%)	0.75
Body surface area (m^2^)	1.93±0.26	1.93±0.26	1.79±0.20	<0.001
Hypertension, n (%)	230 (32%)	230 (34%)	0 (0%)	<0.001
Hyperlipidemia, n (%)	179 (25%)	179 (26%)	0 (0%)	<0.001
Diabetes mellitus, n (%)	87 (12%)	87 (13%)	0 (0%)	0.01
Smoking, n (%)	48 (7%)	48 (7%)	0 (0%)	0.10
Systolic blood pressure	125±18	125±18	124±16	0.92
Diastolic blood pressure	76±10	76±10	77±11	0.57
Hematocrit (L/L)*	0.40±0.05	0.40±0.05	0.39±0.06	0.39
Etiology/Diagnosis, n (%)				<0.001
Normal CMR, n (%)	40 (6%)	0 (0%)	40 (100%)	
Hypertrophic cardiomyopathy, n (%)	175 (24%)	175 (26%)	0 (0%)	
Dilated cardiomyopathy, n (%)	74 (10%)	74 (11%)	0 (0%)	
Myocarditis, n (%)	70 (10%)	70 (10%)	0 (0%)	
Infiltrative, n (%)	30 (4%)	30 (4%)	0 (0%)	
Ischemic, n (%)	22 (3%)	22 (3%)	0 (0%)	
Other non-ischemic cardiomyopathy, n (%)	307 (43%)	307 (45%)	0 (0%)	

Variables are presented as mean ± standard deviation for continuous variables and number with percentage in parentheses for categorical variables. *CMR* cardiovascular magnetic resonance

* Hematocrit was available in 504 (70%) of subjects (483 patients and 21 controls)

**Table 2:** Cardiac MRI parameters

	**All Patients** **(n=718)**	**Cardiomyopathy Patients** **(n=678)**	**Controls** **(n=40)**	**p-value**
1.5T MRI, n (%)	344 (48%)	327 (48%)	17 (43%)	0.52
LV EDVi (mL/m^2^)	88±28	89±28	80±15	0.06
LV ESVi (mL/m^2^)	43±27	44±28	33±8	0.02
LV EF (%)	54±10	53±10	58±3	0.002
LV Mi (g/m^2^)	64±23	65±23	53±13	0.003
RV EDVi (mL/m^2^)	87±30	88±30	82±20	0.35
RV ESVi (mL/m^2^)	45±30	45±31	37±13	0.20
RV EF (%)	52±10	51±10	56±7	0.05
LA size (cm^2^)	25±8	25±8	21±6	0.02
RA size (cm^2^)	20±6	20±6	19±5	0.28
LGE presence, n (%)	366 (54%)	366 (57%)	0 (0%)	<0.001
Myocardial native T1 (ms) at 1.5T*	1038±67	1039±68	1013±35	0.09
Myocardial native T1 (ms) at 3T*	1258±63	1260±64	1220±32	0.003
Myocardial native T2 (ms) at 1.5T*	46.6±3.9	46.6±3.9	46.0±2.9	0.56
Myocardial native T2 (ms) at 3T*	39.1±3.4	39.1±3.5	39.0±2.7	0.87
RV BP T2 (ms) at 1.5T*	139±24	139±24	141±14	0.63
RV BP T2 (ms) at 3T*	70±13	70±13	78±11	0.004
LV BP T2 (ms) at 1.5T*	184±33	184±32	182±28	0.83
LV BP T2 (ms) at 3T*	121±22	121±22	119±20	0.70
RV/LV BP T2 ratio at 1.5T*	0.77±0.14	0.77±0.14	0.79±0.11	0.10
RV/LV BP T2 ratio at 3T*	0.59±0.13	0.59±0.13	0.67±0.09	0.003

Note — Variables are presented as mean ± standard deviation for continuous variables and number with percentage in parentheses for categorical variables

*BP* blood pool, *EDVi* indexed end-diastolic volume, *ESVi* indexed end-systolic volume, *EF* ejection fraction, *LA* left atrium, *LV* left ventricle, *LGE* late gadolinium enhancement, *Mi* indexed mass, *RA* right atrium, *RV* right ventricle

* Subgroup with CMR at 1.5T (344) or 3T (374)

### 3.2 Major adverse cardiac events

Over a median follow-up of 3.2 years (IQR 2.3, 3.8), 53 patients (7%) experienced MACE (event rate 2%/year). All events occurred in the cardiomyopathy cohort. Non-mutually exclusive events included 10 sudden cardiac deaths, 15 cardiac deaths, 3 appropriate ICD discharges, and 34 hospitalizations for heart failure requiring escalation of therapy.

### 3.3 Field strength and sequence effects on blood pool T2 and RV/LV BP T2 ratio

Blood pool T2 values varied markedly by field strength, with substantially higher values at 1.5T than 3T. T2 relaxation times of the RV BP were 81%–99% higher at 1.5T compared to 3T (141±14 ms vs. 78±11 ms, p<0.001 in controls and 139±24 ms vs. 70±13 ms, p<0.001 in cardiomyopathy patients). LV BP T2 was 52%–53% higher at 1.5T versus 3T (182±28 ms vs. 119±20 ms, p<0.001 in controls and 184±32 ms vs. 121±22 ms, p<0.001 in cardiomyopathy patients). Consequently, the RV/LV BP T2 ratio was 18%–31% higher at 1.5T vs 3T (0.79±0.11 vs. 0.67±0.09, p<0.001 in controls and 0.77±0.14 vs. 0.59±0.13, p<0.001 in cardiomyopathy patients), establishing field strength as a dominant determinant of the ratio.

In the group of 15 controls who underwent T2 mapping with both SSFP and FLASH-based technique at 3T, myocardial T2 values were slightly lower with FLASH- compared to SSFP-based T2 maps (38.5±1.8 ms vs. 39.3±2.5 ms, p=0.048). However, there was no difference in RV BP T2 (77±7 ms vs. 77±8 ms, p=0.44), LV BP T2 (122±19 ms vs. 123±21 ms, p=0.58), or RV/LV BP T2 ratio (0.64±0.08 vs. 0.64±0.09, p=0.87) between FLASH- and SSFP-based T2 mapping sequences.

### 3.4 Sex differences of blood pool T2 and RV/LV BP T2 ratio

In addition to field strength, BP T2 values showed modest but consistent sex-related differences, with higher values in women than in men. Compared to men, women had 7%–11% higher RV BP T2 (146±24 ms vs. 132±22 ms, p<0.001 at 1.5T and 73±13 ms vs. 68±13 ms, p<0.001 at 3T) and 4%–7% higher RV/LV BP T2 ratio (0.78±0.14 vs. 0.75±0.13, p=0.03 at 1.5T and 0.62±0.13 vs. 0.58±0.13, p=0.009 at 3T). LV BP T2 values were 6% higher in women compared to men at 1.5T (189±33 ms vs. 178±33 ms, p=0.003) but did not differ significantly at 3T (122±23 ms vs. 120±21 ms, p=0.55).

### 3.5 Prognostic association with MACE

**Fig. 2:**
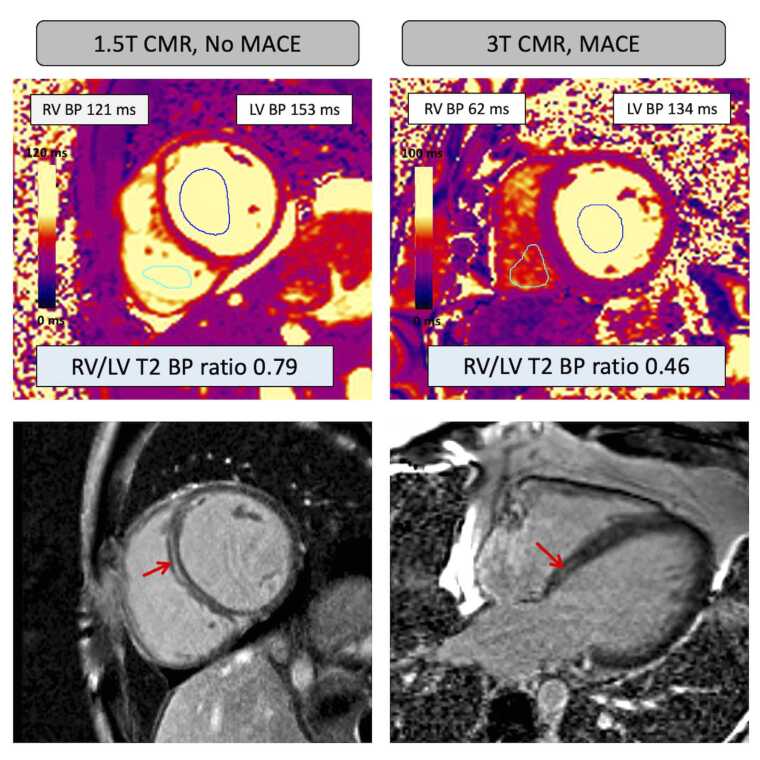
Right ventricular blood pool (teal contour) left ventricular blood pool (blue contour), and RV/LV blood pool T2 ratios. Left panel, 1.5T CMR in a 20-year-old male with dilated cardiomyopathy who did not experience MACE with linear mid-wall LGE at the septum (red arrow) and RV/LV BP ratio of 0.79. Right panel, 3T CMR in a 35-year-old male with dilated cardiomyopathy who experienced MACE with linear mid-wall LGE at the septum (red arrow) and RV/LV BP ratio of 0.46. *CMR* cardiovascular magnetic resonance, *BP* blood pool, *RV* right ventricle, *LV* left ventricle, *LGE* late gadolinium enhancement, *MACE* major adverse cardiovascular event

Patients who experienced MACE had significantly lower RV BP T2 (117±20 ms vs. 141±23 ms, p<0.001 at 1.5T and 64±15 ms vs. 70±13 ms, p=0.02 at 3T) and lower RV/LV BP T2 ratio (0.66±0.13 vs. 0.78±0.13, p<0.001 at 1.5T and 0.50±0.11 vs. 0.60±0.13, p<0.009 at 3T) compared to those who did not experience MACE (Fig. 2). However, LV BP T2 did not differ significantly between those with and without MACE (181±34 ms vs. 184±33 ms, p=0.62 at 1.5T and 127±20 ms vs. 120±22 ms, p=0.15 at 3T).

Each 0.1 unit decrease in the RV/LV BP T2 ratio was associated with a 42% increase in the hazard of MACE in univariable analysis (hazard ratio [HR] 0.58, 95% confidence interval [CI] 0.46, 0.72, p<0.001 at 1.5T and HR 0.58, 95%CI 0.42, 0.80, p=0.001 at 3T). In adjusted models, each 0.1 unit decrease in the RV/LV BP T2 ratio was associated with 41%–42% increase in the hazard of MACE (adjusted HR 0.59, 95%CI 0.45, 0.78, p<0.001 at 1.5T and adjusted HR 0.58, 95%CI 0.40, 0.85, p=0.005 at 3T) with incremental prognostic value beyond patient age, sex, LGE, and cardiomyopathy etiology in nested models (χ^2^[*df* = 1] 13.2, *P*<0.001 at 1.5T and χ^2^[*df* = 1] 8.3, *P*=0.004 at 3T). In fully adjusted multivariable models in the subset of patients with data on hematocrit, RV/LV BP T2 ratio remained a significant predictor of MACE (adjusted HR 0.72, 95%CI 0.52, 0.98, p=0.035 at 1.5T and adjusted HR 0.56, 95%CI 0.36, 0.87, p=0.010 at 3T).

Similarly, each 10 ms decrease in RV BP T2 was associated with a 35%–37% increase in the hazard of MACE in univariable analysis (HR 0.63, 95%CI 0.54, 0.73, p<0.001 at 1.5T and HR 0.65, 95%CI 0.47, 0.92, p=0.013 at 3T), and a 32–35% increase in the hazard of MACE in multivariable analysis (HR 0.65, 95%CI 0.54, 0.78, p<0.001 at 1.5T and HR 0.68, 95%CI 0.47, 0.99, p=0.043 at 3T) with incremental prognostic value beyond patient age, sex, LGE, and cardiomyopathy etiology in nested models (χ^2^[*df* = 1] 22.3, p<0.001 at 1.5T and χ^2^[*df* = 1] 4.3, p=0.04 at 3T). In fully adjusted multivariable models in the subset of patients with data on hematocrit, RV BP T2 remained a significant predictor of MACE (adjusted HR 0.69, 95%CI 0.56, 0.86, p=0.001 at 1.5T and adjusted HR 0.65, 95%CI 0.44, 0.96, p=0.032 at 3T). Conversely, the relationship between LV BP T2 and MACE was not significant (HR 0.97, 95%CI 0.86, 1.08, p=0.58 at 1.5T and HR 1.12, 95%CI 0.95, 1.32, p=0.18 at 3T).

### 3.6 Risk stratification and thresholds

**Fig. 3:**
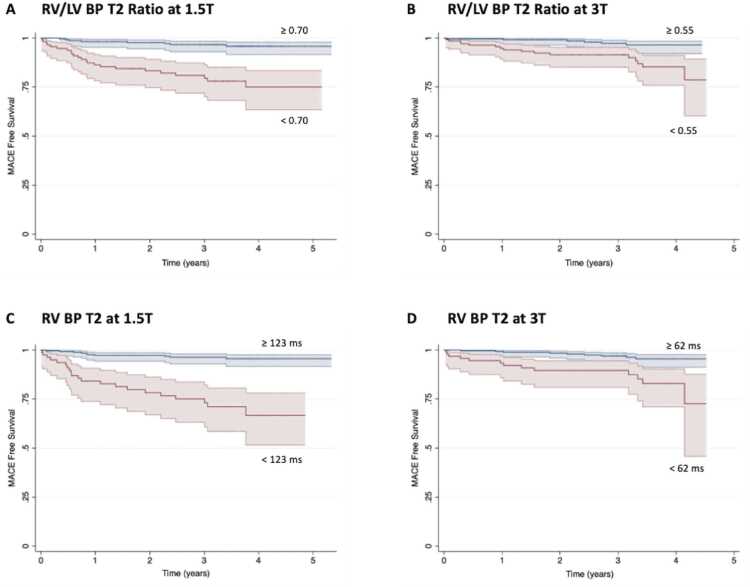
Kaplan-Meier curves for major adverse cardiac event (MACE)–free survival at field strength-specific cutoff points with 95% CIs (shaded). (A) Kaplan-Meier survival curves for 1.5T CMR, using a RV/LV T2 BP ratio cut-point of <0.70. (B) Kaplan-Meier survival curves for 3T CMR, using a RV/LV T2 BP ratio cut-point of <0.55. (C) Kaplan-Meier survival curves for 1.5T CMR, using an RV BP T2 cut-point of <123 ms. (D) Kaplan-Meier survival curves for 3T CMR, using an RV BP T2 cut-point of <62 ms. Patients with RV/LV T2 BP ratios of <0.55 at 1.5T and <0.70 at 3T and RV BP T2 values <123 ms at 1.5T and <62 ms at 3T had worse event-free survival compared with patients with larger values (p<.001 for all). *CMR* cardiovascular magnetic resonance, *CI* confidence interval, *BP* blood pool, *RV* right ventricle, *LV* left ventricle, *MACE* major adverse cardiovascular event, *RV/LV T2 BP* ratio of RV to LV blood pool T2

Optimal cut-points for prediction of 3-year MACE were field strength-specific. An RV/LV BP T2 ratio cut-point of <0.70 at 1.5T had 71% (95%CI 66%, 76%) specificity and 74% (95%CI 55%, 88%) sensitivity (AUC 0.727, 95%CI 0.645, 0.809) and a cut-point of <0.55 at 3T had 65% (95%CI 60%, 70%) specificity and 73% (95%CI 50%, 89%) sensitivity (AUC 0.689, 95%CI 0.590, 0.787). A RV BP T2 cut-point of <123 ms at 1.5T had 81% (95%CI 76%, 85%) specificity and 68% (95%CI 49%, 83%) sensitivity (AUC 0.744, 95%CI 0.658, 0.831), and a cut-point of <62 ms at 3T had 77% (95%CI 72%, 81%) specificity and 59% (95%CI 36%, 79%) sensitivity (AUC 0.681, 95%CI 0.573, 0.788). AUCs did not differ significantly between RV/LV BP T2 ratio and RV BP T2 at 1.5T (χ^2^[*df* = 1] 0.13, *P*=0.72) or 3T (χ^2^[*df* = 1] 0.03, p=0.87). Similarly, AUCs did not differ between field strength for RV/LV BP T2 ratio (χ^2^[*df* = 1] 0.34, p=0.56) or RV BP T2 (χ^2^[*df* = 1] 0.83, p=0.36). These cut-points stratify patients into distinct risk groups with significantly worse event-free survival in patients below these thresholds at both field strengths (p<0.001 for all) (Fig. 3).

### 3.7 Association with CPET parameters

In the subset of 150 patients with CPET within one year of CMR (1.5T in 91 and 3T in 59), RV/LV BP T2 ratio and RV BP T2 modestly correlated with impaired functional capacity (reduced percent predicted oxygen uptake (VO_2_, RV/LV BP T2 ratio: r= 0.37, p<0.001 at 1.5T and r= 0.31, p=0.03 at 3T; RV BP T2: r= 0.50, p<0.001 at 1.5T and r= 0.38, p=0.005 at 3T) and autonomic recovery with impaired heart rate recovery (heart rate drop at 1 min of rest, RV/LV BP T2 ratio: r=0.27, p=.008 at 1.5T and r=0.39, p=0.002 at 3T; RV BP T2: r= 0.22, p=0.03 at 1.5T and r= 0.30, p=0.03 at 3T), Table 3. RV/LV BP T2 ratio correlated with higher ventilatory efficiency (VE/VCO_2_) at anaerobic threshold (r=-0.26, p=.02 at 1.5T and r= −0.52, p<0.001 at 3T), whereas the association with RV BP T2 was only significant at 3T (r=-0.16, p=.15 at 1.5T and r= −0.63, p<0.001 at 3T).

**Table 3:** Correlation of RV/LV T2 Blood Pool Ratio with cardiopulmonary exercise test parameters

**CPET parameter**	**RV/LV T2 Blood Pool Ratio**				**RV Blood Pool T2**			
	**1.5 T (n=91)**		**3 T (n=59)**		**1.5 T (n=91)**		**3 T (n=59)**	
	**r-value**	**p-value**	**r-value**	**p-value**	**r-value**	**p-value**	**r-value**	**p-value**
Resting FVC (L)	0.12	0.27	0.22	0.12	-0.07	0.50	0.34	0.01
Resting FEV1 (L/s)	0.09	0.43	0.24	0.10	-0.09	0.39	0.35	0.01
Exercise time (s)	0.14	0.19	0.07	0.58	0.14	0.18	0.21	0.10
Heart rate recovery (heart rate drop at 1 min of rest, HRR)	0.27	0.008	0.39	0.002	0.22	0.03	0.30	0.03
Peak oxygen uptake (VO2) (mL/kg per min)	0.17	0.12	0.45	0.001	0.09	0.39	0.57	<0.001
Percept predicted oxygen uptake (%ppVO2)	0.37	<0.001	0.31	0.03	0.50	<0.001	0.38	0.005
Slope Ventilatory Efficiency VE/VCO2	-0.06	0.70	-0.09	0.65	-0.10	0.51	-0.36	0.04
Ventilatory Efficiency (VE/VCO2) at anaerobic threshold (AT)	-0.26	0.02	-0.52	<0.001	-0.16	0.15	-0.63	<0.001
Percent measured peak oxygen uptake	-0.23	0.04	-0.09	0.53	-0.36	<0.001	-0.16	0.24
Peak Respiratory Exchange Ratio (RER)	0.06	0.57	0.14	0.32	0.04	0.69	0.08	0.56
Oxygen Uptake Efficiency Slope (OUES)	0.03	0.77	0.13	0.45	-0.08	0.50	0.17	0.32

Note — Data are Spearman correlation coefficients (r-values) of RV/LV T2 blood pool ratio with cardiopulmonary exercise parameters.

*AT* anaerobic threshold, *CPET* cardiopulmonary exercise test, *FEV* Forced expiratory volume, *FVC* Forced vital capacity, *HRR* Heart rate recovery, *OUES* oxygen uptake efficiency slope, *RER* Respiratory exchange ratio, *VCO2* carbon dioxide production, *VE* minute ventilation, *VO2* oxygen uptake

### 3.8 Reproducibility

Intra- and inter-observer agreement were excellent for RV/LV BP T2 ratio (ICC 0.98, 95% CI 0.96, 0.99, and ICC 0.95, 95% CI 0.93, 0.97, respectively), RV T2 BP (ICC 0.99, 95% CI 0.98, 0.99, and ICC 0.97, 95% CI 0.95, 0.98, respectively), and LV T2 BP (ICC 0.93, 95% CI 0.86, 0.96, and ICC 0.96, 95% CI 0.94, 0.98, respectively).

## 4 Discussion

CMR right-to-left ventricular blood pool T2 mapping ratio is an emerging, non-invasive biomarker of intracardiac blood oxygenation gradients [9], [10], [15], [16]. While prior studies have demonstrated associations with adverse outcomes, key uncertainties remain regarding technical determinants, sex-based differences, and physiologic validity. In this cohort of 678 patients with cardiomyopathy and 40 healthy controls, we demonstrate that the RV/LV BP T2 ratio is strongly influenced by MRI field strength, modestly affected by sex, highly reproducible, closely associated with objective measures of cardiopulmonary performance, and independently predictive of major adverse cardiac events. Critically, we establish field strength-specific prognostic cut-points for clinical interpretation, directly addressing a significant barrier to broader clinical implementation.

These findings extend the existing literature in several ways. Most notably, we demonstrate a pronounced field-strength effect, with RV/LV BP T2 ratios 18%–31% higher at 1.5T than at 3T. This magnitude of difference has not previously been reported. Emrich et al. reported pooled ratios of 0.71 in healthy volunteers and 0.89 in patients with left-to-right shunts but did not stratify by field strength [9]. Halfmann et al. evaluated heart failure patients exclusively at 3T, with values closely aligned with our 3T cohort (0.74 in controls and 0.50–0.54 in heart failure patients) [10]. In contrast, Ma et al. evaluated patients exclusively at 1.5T and reported higher mean ratios (0.87 in controls and 0.73 in heart failure), consistent with our 1.5T cohort [15]. Yang et al. reported the only prior comparison across field strengths but included only 20 healthy volunteers and observed minimal differences (0.83±0.06 vs. 0.82±0.06), possibly due to limited power and methodological heterogeneity [16]. By demonstrating consistent and substantial differences across field strengths in over 700 patients, our study provides robust evidence that the RV/LV BP T2 ratio is not inherently field-independent and that pooling values across scanners obscures clinically meaningful variation.

This field-strength dependence is supported by established biophysical principles. Myocardial and blood-pool T2 values decrease with increasing field strength due to heightened susceptibility-related dephasing [11], [26]. T2 relaxation also shortens as the concentration of deoxygenated hemoglobin increases, and in vitro studies demonstrate that the slope of T2 reduction with declining oxygen saturation becomes progressively steeper at higher magnetic field strengths [27], [28], [29], [30]. Because venous blood contains more deoxyhemoglobin than arterial blood, RV blood is intrinsically more susceptible to these field-dependent BOLD effects [5], [31]. Consequently, RV blood pool T2 decreases more sharply than LV blood pool T2 at 3T, compressing the RV/LV BP T2 ratio relative to 1.5T. Clinical data further support this mechanism, as RV BP T2 and RV/LV BP T2 ratios track hemodynamic severity and improve following therapies that increase venous oxygenation in pulmonary hypertension [8], [14], [32]. Flow-dependent signal effects on the blood pool are more pronounced at higher field strengths and may have a larger impact on left ventricular BP measurements due to higher flow velocities and more complex flow patterns [33]. Together, these observations provide a coherent mechanistic explanation for the higher RV/LV BP T2 ratios observed at 1.5T compared to 3T and underscore the need for field strength-specific calibration and thresholds.

We also observed modest but consistent sex-related differences, with RV/LV BP T2 ratios 4%–7% higher in women compared to men. To our knowledge, previous studies have not reported sex-stratified values, making this an incremental and potentially clinically relevant finding. Women have higher resting arterial oxygen saturation, lower hemoglobin concentrations, and distinct cardiopulmonary mechanics and ventilatory efficiency compared with men, all of which may influence venous oxygenation and associated BP T2 values [34], [35], [36], [37]. Although smaller in magnitude than the effect of field strength, these differences may be relevant and warrant further investigation in larger sex-balanced cohorts.

Our results also confirm and extend prior prognostic data. Two prior studies demonstrated that lower RV/LV BP T2 ratios independently predict adverse outcomes in patients with dilated cardiomyopathy and heart failure [15], [16]. We observed similar effect sizes but in a more diverse cohort with adjustment for age, sex, LGE, cardiomyopathy etiology, and hematocrit. Adjustment for hematocrit is relevant given its inverse relationship to native T1 and T2 blood pool values [38]. We also established field strength-specific thresholds for MACE prediction, demonstrating that a single universal cut-point is inappropriate. This represents a critical step toward clinical translation by providing actionable thresholds aligned with real-world scanner variability.

A unique contribution of this study is the integration of CPET, providing physiologic validation of this imaging metric. Lower RV/LV BP T2 ratios were associated with reduced functional capacity, impaired ventilatory efficiency, and delayed autonomic recovery, all established indicators of reduced cardiopulmonary reserve and poor prognosis in patients with heart failure [39], [40]. These findings align with known pathophysiologic consequences of venous desaturation and extend prior observations based on 6-minute walk testing [5], [10], [31], [41]. This data supports the interpretation that the RV/LV BP T2 ratio reflects integrated cardiopulmonary physiology rather than a purely technical imaging phenomenon and suggests that a lower RV/LV BP T2 ratio may be an early imaging signal of impaired functional exercise capacity. In patients with heart failure, lower RV/LV BP T2 ratio may be related to impaired oxygen delivery and higher peripheral blood desaturation [10].

Reproducibility was excellent and consistent with prior reports [9], [10], [14]. Interestingly, absolute RV BP T2 demonstrated similar prognostic and physiological performance to the RV/LV BP T2 ratio. While the ratio may partially contextualize venous oxygenation relative to the systemic background and other sources of variability, absolute RV BP T2 offers practical advantages as an opportunistic imaging biomarker, including simplicity, efficiency, and ease of automation, aligning with strategies to maximize the clinical value and efficiency of CMR without increasing scan time or complexity [42], [43], [44], [45], [46]. The RV/LV BP T2 ratio may still provide complementary value in settings where blood pool T2 values are globally shifted, such as anemia, or in multi-center studies with greater sequence heterogeneity [26], [27], [28], [47]. With either metric, interpretation should be grounded in field strength-specific reference ranges.

## 5 Limitations

This study has limitations. Its single-center retrospective design may limit generalizability, and vendor differences were not evaluated. T2 mapping sequences differed between MRI units; however, all mapping data were analyzed stratified by field strength and specific sequence. Paired analysis in a group of 15 controls demonstrated that myocardial T2 values with FLASH readout were slightly lower compared to SSFP readout, consistent with prior literature [18], [48]. However, absolute RV and LV BP, and RV/LV BP T2 ratio, did not differ between techniques, indicating that differences in T2 mapping sequence do not explain the observed field strength differences. Paired evaluation of both T2 mapping techniques was only available on 3T and comparison of techniques at 1.5T could be evaluated in a future study. Although the cohort was large, the number of outcome events was limited, restricting subgroup analysis. CPET was not contemporaneous with CMR in all patients, with an interval of up to one year. Finally, although sex differences were observed, subgroup sizes were insufficient to define reliable sex-specific thresholds.

## 6 Conclusions

In conclusion, the RV/LV BP T2 ratio demonstrates strong dependence on MRI field strength, reflects physiologically meaningful cardiopulmonary impairment, and independently predicts major adverse cardiac events with high reproducibility. These findings establish a mechanistic and clinical foundation for broader application and highlight the need for field strength-specific thresholds. Ventricular blood pool T2 metrics have the potential to enhance functional characterization and risk stratification in routine CMR practice. Lower RV/LV BP T2 ratio and RV BP T2 could be used to non-invasively identify patients at higher risk of adverse events who may benefit from closer monitoring and more aggressive up-titration of guideline-directed therapy.

## Author contributions

**Moran Drucker Iarovich:** Writing – original draft, Methodology, Investigation, Formal analysis, Data curation. **Fadi Ibrahim:** Writing – review & editing, Data curation. **Joao Francisco Matos:** Writing – review & editing, Data curation. **Nilushide Silva:** Data curation. **William Holden Lowes:** Investigation, Data curation. **Yasbanoo Moayedi:** Resources, Investigation, Data curation, Conceptualization. **Paaladinesh Thavendiranathan:** Writing – review & editing, Methodology, Investigation. **Tilman Emrich:** Methodology, Investigation, Conceptualization. **Akos Varga-Szemes:** Writing – review & editing, Conceptualization. **Kate Hanneman:** Writing – review & editing, Supervision, Project administration, Methodology, Investigation, Formal analysis, Data curation, Conceptualization.

## Declaration of competing interest

The authors declare that they have no known competing financial interests or personal relationships that could have appeared to influence the work reported in this paper.

## Footnotes

Appendix A Supplementary data associated with this article can be found in the online version at doi:10.1016/j.jocmr.2026.102760.
