# Supplementary material for: Field strength dependence, physiologic correlates, and prognostic significance of ventricular blood pool T2 mapping on cardiovascular magnetic resonance imaging
Source: J Cardiovasc Magn Reson. 2026 Jun 6;28(2):102760. doi: 10.1016/j.jocmr.2026.102760 (PMC13292778; doi:10.1016/j.jocmr.2026.102760)
Supplement: Supplementary file 1 — Supplementary material [file mmc1.docx]

**Supplemental Table S1.** T2 Mapping Parameters

|  | 1.5T | 3T |
| --- | --- | --- |
| Sequence | T2-prepared SSFP | T2-prepared FLASH |
| Bandwidth | 1184 Hz/Px | 1184 Hz/Px |
| Parallel Imaging | GRAPPA; R=2 | GRAPPA; R=2 |
| Base Resolution | 192 | 192 |
| Pixel Dimensions (mm) | 1.9 x 1.9 | 1.9 x 1.9 |
| Slice thickness (mm) | 8 | 8 |
| TR/TE (ms) | 2.5/1.1 | 3.1/1.3 |
| Flip angle (˚) | 70 | 35 |
| Other | Effective TR=3RR  T2 prep 0/25/55ms | Effective TR=3RR  T2 prep 0/30/55ms |
| FLASH = Fast Low Angle SHot; GRAPPA = GeneRalized Autocalibrating Partial Parallel Acquisition; SSFP = Steady State Free Precession. | | |

**Supplemental Table S2. Key cardiopulmonary exercise test parameters**

| **VARIABLE** | **DESCRIPTION** |
| --- | --- |
| *Resting Forced Vital Capacity (FVC)* | The total volume of air that can be exhaled during a maximal forced expiration effort |
| *Resting Forced expiratory volume in one second (FEV_1_)* | The volume of air exhaled in the first second under force after a maximal inhalation |
| *Exercise time* | Total duration of exercise in seconds |
| *Heart rate recovery (heart rate drop at 1 minute of rest, HRR)* | The drop in heart rate in the first minute after stopping exercise |
| *Oxygen Uptake (VO_2_)* | The amount of oxygen an individual can utilize in cellular metabolism |
| *Peak Oxygen Uptake (pVO_2_)* | Highest VO_2_ achieved during maximal effort. Objective indicator of exercise capacity based on peak oxygen uptake during maximal exercise |
| *Percent Predicted pVO_2_ (%ppVO_2_)* | The expected pVO_2_ adjusted for age, gender, and weight according to Wasserman-Hansen equation |
| *Ventilatory Efficiency (VE/VCO_2_)* | Ratio of how many ventilatory litres (gas exchange) it takes to remove 1 litre of CO_2_ from the body |
| *Ventilatory Efficiency at Anaerobic Threshold (AT)* | Indicates switch from aerobic to anaerobic metabolism causing a divergence in VE relative to VO_2_ |
| *Respiratory Exchange Ratio (RER)* | Ratio of gas exchange used as a measure of volitional effort during exercise based on VCO_2_ produced/VO_2_ consumed |
| *Oxygen Uptake Efficiency Slope (OUES)* | Relation between VO2 (ml/min) and log minute ventilation (VE; L/min) |
